# Supplementary material for: Long-term alterations of collagen reconstruction and basement membrane regeneration after corneal full-thickness penetrating injury in rabbits
Source: PLoS One. 2025 Apr 24;20(4):e0320802. doi: 10.1371/journal.pone.0320802 (PMC12021247; doi:10.1371/journal.pone.0320802)
Supplement: Attachment — Submitted filename: Review.pdf [file pone.0320802.s001.pdf]

Li and colleagues presented a very interesting research investigating the long-term alterations of collagen reconstruction and basement membrane (BM) regeneration after corneal full-thickness penetrating injury in rabbits.

Nice work and important contributions to the field.
A few relatively minor comments/questions/suggestions:

Introduction, Line 73: please describe more about the cytokines: TGFbeta, PDGF and how they participate regulating the wound healing and myofibroblasts differentiation and fibrosis generation

Material and Methods, line 91: please describe this surgery better, what was the size of injury? were suture places? any adverse events during or after surgery?

Material and methods, line 97-99 “General anesthesia was achieved through intravenous injection of pentobarbital sodium (3 ml/kg) via an auricular vein, followed by topical anesthesia with 0.5% proparacaine hydrochloride eye drops
Material and methods, Line 106-108: “Following general anesthesia, euthanasia was performed using an intravenous injection of 100 mg/kg pentobarbital sodium"

Was pentobarbital used for anesthesia ou euthanasia??
Please correct this information in the text

Discussion, line 422: Authors could add in the discussion previous data from papers that showed EBM and DM role and myofibroblasts differentiation after alkali chemical burn injuries in rabbits

Discussion, line 441 and 469: In rabbits the endothelium is also different - endothelium cells may regenerate in rabbits

Conclusion, line 496 / and abstract, line 39 (conclusion abstract) : “Our findings suggest that Col IVα6 plays a regulatory role in corneal fibrosis”—> with the information the paper gave it’s not possible to state this, better to use “appears to be involved in regulating corneal fibrosis” as used in the text

Also please add about the cytokines role - and basement membranes relation with them and myofibroblast differentiation
