# Supplementary material for: Long-term alterations of collagen reconstruction and basement membrane regeneration after corneal full-thickness penetrating injury in rabbits
Source: PLoS One. 2025 Apr 24;20(4):e0320802. doi: 10.1371/journal.pone.0320802 (PMC12021247; doi:10.1371/journal.pone.0320802)
Supplement: Attachment — Submitted filename: Response_to_Reviewers_auresp_3.docx [file pone.0320802.s003.docx]

**Response Letter**

Dear Editors and Reviewers:

We sincerely appreciate the time and effort dedicated by the reviewers and the editorial team to evaluate our manuscript titled “Long-term alterations of collagen reconstruction and basement membrane regeneration after corneal full-thickness penetrating injury in rabbits”(PONE-D-24-34892R2). We have carefully addressed all comments raised during the second round of review. The revised manuscript incorporates all suggested changes, and detailed point-by-point responses are provided below.

**Response to Academic Editor’s Comments**:

**Comments：**The authors appear to have misunderstood the cited papers on nidogen-2 and type IV collagen. They have corrected an erroneous statement in previous version with another erroneous one: “The matrix components in this model, including nidogen-2 (detected using an antibody not validated for rabbit tissue) and type IV collagen, exhibit altered localization patterns compared to their physiological distribution in human corneal basement membranes. Specifically, nidogen-2 in the fibrotic stroma may reflect non-specific antibody binding or pathological stromal deposition, while type IV collagen is abnormally organized and lacks integration with the native basement membrane structures. ^[29, 39]^ Consequently, this distinct composition hinders the regenerative capacity of stromal keratocytes and fibroblasts”.

1. The cited papers did not deal with type IV collagen at all.

2. It is unnecessary to specify that the antibody was not validated in the studied species.

3. It is unclear what the pattern alteration was.

4. Nidogen-2 (and nidogen-1) is expressed in the stroma, thus, it is unclear what pathology the authors refer to.

5. Again, type IV collagen statement is made up as no such component was studied in the cited papers.

6. The statement about “hindering the regenerative capacity of stromal keratocytes and fibroblasts” is purely speculative and is not supported by data. Overall, the quoted sentences should be altogether removed as misleading, and the authors more carefully read the references.

**Response**: We sincerely appreciate the editor's suggestions. We acknowledge that some omissions may have occurred in our literature review, and we will ensure a more comprehensive analysis in future work. As suggested, we have removed the indicated sentence from the forth paragraph of the Discussion section. This deletion improves the conciseness of the argument without affecting the overall logic of the section.

We believe these revisions have significantly improved the manuscript and addressed all reviewers’ concerns. We are happy to provide further modifications if needed. Thank you again for your time and valuable feedback.

Yours sincerely,

Xia Li

MD, PHD

Department of Opthalmology, The First Affiliated Hospital of Guangxi Medical University, 6# Shuangyong Road, Nanning, Guangxi, China

E-mail: lixiagmu066@163.com
